# Responses in Micro-Mineral Metabolism in Rainbow Trout to Change in Dietary Ingredient Composition and Inclusion of a Micro-Mineral Premix

**DOI:** 10.1371/journal.pone.0149378

**Published:** 2016-02-19

**Authors:** P. Antony Jesu Prabhu, Inge Geurden, Stéphanie Fontagné-Dicharry, Vincent Veron, Laurence Larroquet, Catherine Mariojouls, Johan W. Schrama, Sadasivam J. Kaushik

**Affiliations:** 1 Institut National de la Recherche Agronomique (INRA), UR1067, Nutrition, Metabolism and Aquaculture (NuMeA), Aquapôle INRA, 64310, Saint-Pée-sur-Nivelle, France; 2 AgroParisTech, Paris Institute of Technology for Life, Food and Environmental Sciences, 16 rue Claude Bernard, 75231, Paris, Cedex 5, France; 3 Aquaculture and Fisheries Group, Wageningen Institute of Animal Sceinces (WIAS), Wageningen University, PO Box 338, 6700 AH, Wageningen, The Netherlands; Oklahoma State University, UNITED STATES

## Abstract

Responses in micro-mineral metabolism to changes in dietary ingredient composition and inclusion of a micro-mineral premix (Fe, Cu, Mn, Zn and Se) were studied in rainbow trout. In a 2 x 2 factorial design, triplicate groups of rainbow trout (initial weight: 20g) were fed over 12 weeks at 17°C a fishmeal-based diet (M) or a plant-ingredient based diet (V), with or without inclusion of a mineral premix. Trout fed the V vs. M diet had lower feed intake, growth, hepato-somatic index, apparent availability coefficient (AAC) of Fe, Cu, Mn and Zn and also lower whole body Se and Zn concentration, whereas whole body Fe and Cu and plasma Fe concentrations were higher. Feeding the V diet increased intestinal ferric reductase activity; at transcriptional level, hepatic hepcidin expression was down-regulated and ferroportin 1 was up-regulated. Transcription of intestinal Cu-transporting ATPases and hepatic copper transporter1 were higher in V0 compared to other groups. Among the hepatic metalo-enzyme activities assayed, only Se-dependent glutathione peroxidase was affected, being lower in V fed fish. Premix inclusion reduced the AAC of Fe, Cu and Zn; increased the whole body concentration of all micro- minerals; up-regulated hepatic hepcidin and down-regulated intestinal ferroportin 1 transcription; and reduced the transcription of Cu-transporting ATPases in the intestine. Overall, the regulation of micro-mineral metabolism in rainbow trout, especially Fe and Cu, was affected both by a change in ingredient composition and micro-mineral premix inclusion.

## Introduction

Micro-minerals such as Fe, Cu, Mn, Zn and Se are essential to fish [1,2,3]. A low or a high supply of dietary or aqueous trace minerals affects the associated biochemical and physiological responses in fish [4]. Fish meal, rich in micro-minerals [5], has been the major protein source in feed of farmed fish over the years. However, due to the limited supply, the use of fishery-derived ingredients such as fish meal (FM) in fish feeds, particularly in salmonid feeds, has seen a significant reduction, being replaced to a large extent by plant ingredient sources [6]. In humans, vegetarian diets are reported to be limiting in the supply of bioavailable micro-minerals such as Fe, Zn and Se, but are generally good sources of Cu and Mn. The latter are present at high concentrations in plant ingredients, in contrast with Se which is higher in animal than in plant protein sources [7]. The intrinsic form of the dietary micro-minerals is also important. For instance, heme-bound Fe present in fish or meat based diets is relatively more bioavailable than non-heme Fe present in plant-derived ingredients [8]. It is generally recommended to supplement Fe, Cu, Mn, Zn and Se to fish feeds due to their low and variable levels in practical feed ingredients and also due to possible interactions with other dietary components which may affect their availability [9]. Indeed, secondary metabolites (anti-nutritional factors, ANFs) in plant ingredients may directly or indirectly affect micro-mineral metabolism [10]. The entero-hepato-pancreatic system is the target of major plant ingredient ANFs such as phytic acid, protease inhibitors, lectins, alkaloids [10]. Phytic acid can directly reduce availability of micro-minerals whereas other ANFs may modify uptake and body micro-mineral status by interfering in micro-mineral metabolism at the level of gastrointestinal tract or liver [11]. The underlying cellular and molecular mechanisms by which plant ingredients interact with micro-mineral metabolism in fish remain little explored.

Over the years, various functional proteins and their encoding genes regulating cellular transport of micro-minerals have been characterised in mammals, a few of which have also been identified in teleost species [12]. These include brush border metal reductases and divalent metal transporters (DMTs) for apical uptake of Fe, Cu, Mn or Zn [4]; ferroportin (FPN1) for basolateral extrusion of Fe; hepcidin (HAMP) the iron regulatory peptide; and heme-oxygenase (HO) for heme degradation to release Fe [13,14]; Cu-chaperons (ATOX1 and CCS) and Cu-transporting-ATPases, ATP7a and ATP7b for intra-cellular trafficking of Cu [15,16]. Besides the transporters, activity and expression of metalo-enzymes have been used as markers for micro-mineral status [7]. Most of these markers are shown to respond to exogenous supply of respective micro-minerals. In this scenario, the intriguing questions are, (i) does the transition from fish meal based diets to predominantly plant ingredient based diets affect micro-mineral metabolism in fish?; and (ii) does the supplementation of a micro-mineral premix affect or interact with the ingredient composition of the diet in micro-mineral absorption or metabolism? In order to address these questions, we undertook a 2 x 2 factorial design study with rainbow trout fed either a FM-FO based diet (M) or a totally plant ingredient based (V) diet, with or without micro-mineral (Fe, Cu, Mn, Zn and Se) premix inclusion, and analysed the responses related to absorption, transport or metabolism of micro-minerals.

## Material and Methods

Animal experiments and sampling procedures followed the guidelines of the National Legislation on Animal Care of the French Ministry of Research (Decree no. 2001–464, May 29, 2001) and the animal ethics committee of INRA (INRA 2002–36, April 14, 2002). The INRA experimental facility is certified under the permit number A402281 for animal services and permit number FR40090951-40090002 for animal feed production by the French veterinary services. This study has been approved by the Ethics Committee. The scientist in charge of the experimentation received training and personal authorization (N°B64 11 001).

### Experimental diets

Two basal diets namely M0 (FM-FO based) and V0 (plant-ingredient based) were formulated to meet the protein, energy and macro-mineral requirements of rainbow trout (Table 1). The M0 diet was based on fishmeal (FM) and fish oil (FO), while the V0 diet was made entirely of plant-derived ingredients. Essential micro-minerals (Fe, Cu, Mn, Zn and Se) were supplemented to the basal diets, as a premix at 1% inclusion level, to provide diets M1 and V1, respectively. The premix was formulated to provide Fe, 52.5 mg; Cu, 7.5 mg; Mn, 12 mg; Zn, 14 mg and Se, 0.15 mg per kg diet, at 1% inclusion, on as fed basis. These concentrations are based on data on micro-mineral requirements of rainbow trout [17,18]. The levels of analysed micro-mineral concentrations of the basal and the premix supplemented diets are provided in Table 2.

**Table 1:** Ingredient and chemical composition of the basal diets.

**Ingredients (g/kg diet)**	**M**	**V**
Norwegian herring meal, (CP 70; Sopropêche, France)	625.7	-
Corn gluten meal (CP 60; Inzo, France)	-	180.0
Wheat gluten (CP 70; Roquette, France)	-	200.0
Soybean meal (CP 48; Inzo, France)	-	50.0
Soy protein concentrate (Estrilvo; CP 70; Sopropêche, France)	-	170.0
White lupin meal (Terrena, France)	-	50.0
Extruded peas (Aquatex, Sotexpro, France)	-	30.0
Rapeseed meal (Primor 00; Sud-Ouest Aliment, France)	-	40.0
Whole wheat	245.6	31.8
Soy lecithin (Louis François, France)	-	20.0
L-Lysine (Eurolysine)	-	13.4
L-methionine (Evonik, Germany)	-	3.0
CaHPO4.2H20 (18%P; 22% Ca)	-	21.7
Attractant Mix§	-	15.0
Micro-mineral premix¶	0 or 10	0 or 10
Vitamin premix†	10	10.0
Yttrium oxide (Sigma-Aldrich, USA)	0.1	0.1
Fish oil (southern hemisphere, Sopropêche, France)	118.6	-
Rapeseed Oil (Daudry, France)	-	66.0
Linseed Oil (Daudry, France)	-	66.0
Palm Oil (Daudry, France)	-	33.0
**Analytical composition**		
Dry matter (DM), g/kg	948	949
Crude protein, g/kg DM	494	500
Crude lipid, g/kg DM	221	227
Crude ash, g/kg DM	82	40
Energy, kJ/g DM	23.8	25.1
**Analysed concentration of major minerals (g/kg DM)**		
Phosphorus, g/kg DM	12.9	9.4
Calcium, g/kg DM	11.3	8.0
Ca/P ratio	0.9	0.9
Magnesium, g/kg DM	1.76	0.95
Potassium, g/kg DM	12.2	4.2
Sodium, g/kg DM	9.4	1.4

^§^ Attractant premix: glucosamine, 5g; taurine, 3g; betaine, 3g; glycine, 2g and alanine, 2g.

^†^ Vitamin premix (IU or mg/kg diet): DL-a tocopherol acetate, 60 IU; sodium menadione bisulphate, mg; retinyl acetate, 15,000 IU; DL-cholecalciferol, 3000 IU; thiamin, 15 mg; riboflavin, 30 mg; pyridoxine, 15 mg; B12, 0.05 mg; nicotinic acid, 175 mg; folic acid, 500 mg; inositol, 1000 mg; biotin, 2.5 mg; calcium panthotenate, 50 mg; choline chloride, 2000 mg. (UPAE, INRA).

^¶^ Micro-mineral premix (g/kg premix): FeSO4.7H2O (21% Fe; 11.5% S), 25 g; CuSO4.5H2O (25.45% Cu; 12.8% S), 3 g; MnSO4.H2O (33% Mn; 19% S), 3 g; ZnSO4.H2O (36% Zn; 18% S), 4 g; Na2SeO3 (46% Se; 27% Na), 0.03 g and α-cellulose, 964.93 g

**Table 2:** Analysed dietary concentration of supplemented micro-minerals (mg/kg DM).

Diet code	M0	M1	V0	V1
**Iron (Fe)**	161.6	212.9	153.8	205.4
**Copper (Cu)**	6.9	14	7.0	12.9
**Manganese (Mn)**	9.4	22.7	88.6	100.9
**Zinc (Zn)**	62.8	72.5	42.9	52.5
**Selenium (Se)**	1.09	1.27	0.24	0.40

The NRC [17, 18] recommended minimal dietary inclusion levels (on available basis) for the above listed micro-minerals for rainbow trout are as follows: Fe, 60 mg/kg DM; Cu, 3 mg/kg DM; Mn, 12 mg/kg DM; Zn, 15 mg/kg DM and Se, 0.15 mg/kg DM.

### Experimental animals, design and rearing condition

Rainbow trout juveniles (19.8 ± 0.8 g, initial weight) were distributed into 12 experimental units, each of 500 L (40 fish unit^-1^). Each unit was randomly assigned to one of the four dietary treatments in triplicates following a 2x2 factorial design. The fish were hand fed twice a day to apparent visual satiation for a period of 12 weeks (6 days a week). The fish were reared in flow-through systems at the experimental fish farm (INRA, Donzacq, Landes, France). Water temperature was 17± 0.5°C and the water flow rate was set at 50 L min^-1^ during the experimental period.

### Fish, tissue and faecal sampling

At the end of the 12 week growth trial and 24h after the last meal, a pooled sample of 6 fish from each experimental unit was taken for final body composition analysis. Further, 3 more fish from each experimental unit were randomly withdrawn, anaesthetised (benzocaine, 30 mg L^-1^) and sampled for blood from the caudal vein using heparinised syringe. The blood was then centrifuged (3000g for 5 min) and the recovered plasma were immediately stored at -20°C until mineral analysis. Subsequent to blood sampling, the fish were euthanized by a sharp blow to the head. Liver and anterior intestine (without caeca) were dissected, rinsed in 0.9% NaCl to remove any blood (in liver) or food and faecal remains (in intestine) and immediately frozen in liquid nitrogen and stored at -80°C until further analysis. After the tissue sampling, 25 fish of each experimental unit were used for the determination of apparent availability coefficient (AAC) of minerals. The fish were fed a single meal (86 g tank^-1^) and 9h after the meal, faecal samples were collected by the method of stripping [19]. The samples were collected over ice, frozen immediately and stored at -20°C until mineral analysis.

### Analytical methods

#### Chemical composition

The chemical composition of the diets was analysed by the following methods: dry matter after drying at 105°C for 24h, ash by combustion at 600°C for four hours in a muffle furnace, crude protein (Nx6.25) by Kjeldahl method in acid digested samples, crude lipid by petroleum ether extraction using Soxhlet method (Soxtherm) and gross energy content in an adiabatic bomb calorimeter (IKA, Heitersheim Gribheimer, Germany). The concentrations of Fe, Cu, Mn and Zn in the diets, whole fish, faeces and plasma were analysed using inductively coupled plasma-mass spectrometry (ICP-OES) at USRAVE-INRA, Bordeaux, France. The Se and yttrium content in the samples (except plasma) were analysed using ICP-MS at LCABIE-UPPA, Pau, France.

#### Selection of micro-mineral responsive bio-markers for molecular analysis

Bio-markers known to be involved in absorption, transport and metabolism of Fe and Cu were studied in detail based upon, the similar levels of Fe and Cu in diets M and V (Table 2); inherent differences in the form of Fe between diets M and V; and previous observations of lower endogenous Cu loss when fed diet V [20]. The study on the molecular responses of other micro- minerals Mn, Zn and Se was restricted to the activity or expression of their respective metalo-enzymes such as superoxide dismutase (Mn-SOD and CuZn-SOD); alkaline phosphatase (for Zn) and glutathione peroxidase (for Se), respectively. Details of the primer sequences used for the amplification of the target genes are presented in Table 3.

**Table 3:** Primers used for gene expression analysis by real-time quantitative RT-PCR.

Gene	Accession number (GenBank or SIGENAE)	Primer sequence (5′→3′)	Product size (bp)	Annealing temp. (°C)
EF1α	AF498320.1*	F: TCCTCTTGGTCGTTTCGCTG	159	59
		R: ACCCGAGGGACATCCTGTG		
HAMP	BX088223.s.om.10	F: GGAGGAGGTTGGAAGCATTG	196	59
		R: GATGGTTTTAGTGCAGGCAGG		
HO1	CA387878.s.om.10	F: ACTCTTCCGCAGTACAAGCT	212	59
		R: CTGTGTGTTGCAGCAGGAAT		
FPN1	CA351776.s.om.10	F: GTCCTCTTACTGGGCGCTAT	224	59
		R: GCCAGGTTAGCGATGTTAGC		
Nramp-β	AF048761.1*	F: CACCTCCCCTCCGGCTT	156	60
		R: CCTGGGTCAAGATAGGCGAT		
Nramp-γ	EF495162.1*	F: GCCATCCTCAACAGTGTCT	200	57
		R: CTTTAGCTCCAGACTGTAGATCA		
CTR1	GU723513.1*	F: GTTGTTTCCTGCTGGCTGTG	192	59
		R: GTAACACCGTCTGCAGCAAG		
ATP7a	BX295327.s.om.10	F: CATGCCGGTGACTAAGAAGC	244	59
		R: AATGAGGATCCAGGCGAACA		
ATP7b	FYV3OTN01C7RN9.s.om.10	F: CTGAGATGACTGGGGTGTGT	183	59
		R: GTCTTTGAAGGGGAGGGGTT		
ATOX1	BX300064.s.om.10	F: ATGTGAGGGATGCTCTGGTG	154	59
		R: AGCCTCCTTTCCAGTCTTCT		
SOD1	AF469663.1*	F: TGGTCCTGTGAAGCTGATTG	201	56
		R: TTGTCAGCTCCTGCAGTCAC		
SOD2	CA352127.1*	F: TCCCTGACCTGACCTACGAC	201	57
		R: GGCCTCCTCCATTAAACCTC		
CAT	BX087110.3*	F: TGATGTCACACAGGTGCGTA	195	55
		R: GTGGGCTCAGTGTTGTTGAG		
GPX1b1	CA357669.1*	F: CGAGCTCCATGAACGGTACG	183	59
		R: TGCTTCCCGTTCACATCCAC		
GPX1b2	HE687023*	F: TCGGACATCAGGAGAACTGC	121	56
		R: TCCTTCCCATTCACATCCAC		
GPX4a1	HE687024*	F: GAAAGGCTTCCTGGGAAATG	112	56
		R: CTCCACCACACTGGGATCAT		

*GenBank accession number; F, forward primer; R, reverse primer; EF1α, elongation factor 1α; HAMP, Hepcidin anti-microbial peptide; HO1, heme oxygenase 1; FPN1, ferroportin1; Nramp-β, Natural resistance associated macrophage protein beta polypeptide; Nramp-γ, Natural resistance associated macrophage protein gama polypeptide; CTR1, copper transporter I; ATP7a, Cu^++^ transporting ATPase-alpha polypeptide; ATP7b, Cu^++^ transporting ATPase-beta polypeptide; ATOX1, copper transporter protein ATOX1; SOD1, superoxide dismutase 1; SOD2, superoxide dismutase 2; CAT, catalase; GPX1, glutathione peroxidase 1; GPX4, glutathione peroxidase 4

#### Enzyme assays

Samples of liver and anterior intestine were ground respectively in 8 and 4 volumes of ice cold buffer (TRIS HCl, 50mM; NaCl, 150mM; pH, 4). After homogenization, the samples were subjected to sonic disruption for one minute. The samples were kept on ice during sonication. Homogenates were then centrifuged for 10min at 3500rpm at 4°C and the supernatants were immediately used for enzyme assays. Activities of ferric reductase (FR, EC 1.16.1.7) and cupric reductase (CR, EC 1.16.1) were determined from the same extract. FR activity was measured as described by Mazoch et al. [21], omitting flavin mononucleotide in the mixture. The reduction of iron was followed by the formation of the coloured Fe(II)-ferrozine complex and monitoring the change in absorbance at 562 nm. CR activity was assayed according to Wyman et al. [22], the Cu(I)-bathocuprionedisulfonate complex was monitored by the change in absorbance at 482 nm. ALP activity was measured using a commercial kit (Enzyme ALP, bioMérieux, ref 63509). Antioxidant enzyme activities CAT (EC 1.11.1.6) and GPX (EC 1.11.1.9) were assayed in the liver homogenates following the method of Fontagné et al. [23]. Total SOD (EC 1.15.1.1) activity was measured using a commercial kit (Sigma, St Louis, MO, USA ref 19160-1KT-F); MnSOD (SOD2) was measured by inhibiting CuZn-SOD using 5 mM KCN (Sigma) as specific inhibitor and CuZn-SOD (SOD1) activity was calculated by subtracting Mn-SOD activity from total SOD activity [24]. In all the enzyme assays, the reaction was initiated by the addition of a specific substrate; a blank with water instead of the substrate was run for each sample. One unit of enzyme activity was defined as the amount of enzyme that catalysed the transformation of 1 μmol of substrate per min at 30°C. Protein concentration of all the samples were measured in triplicate by the method of Bradford [25], using a protein assay kit (BioRad, Munich, Germany) with bovine serum albumin as the standard. In all cases, a Power Wave X (BioTek Instrument, Inc.) was used as the plate reader. The enzyme activities were expressed specific to per mg of protein.

#### Gene expression analysis

Total RNA was extracted from liver and anterior intestine samples (n = 9 per treatment) using Trizol reagent (Invitrogen, Cergy-Pontoise, France) as previously described [23]. For quantitative RT-PCR, complementary DNA was generated from 1 μg total RNA using SuperScript^®^ III reverse transcriptase (Invitrogen) with a mix of oligo(dT) and random primers (Promega, Charbonnières, France). RT was performed in duplicate for each sample and the quantitative PCR analyses were performed in LightCycler 480 II thermocycler (Roche) using LightCycler 480 SYBR Green I Master mix (Roche Diagnosis, Indianapolis, IN, USA). Total reaction volume was 6μL, with 2μL of cDNA (RT product) and 4μL of master mix added with 0.4mM of each primer. Relative quantification of target gene transcripts were normalized using Elongation Factor 1α (EF1α) as the reference gene and M0 as the reference group following the method of Pfaffl [26].

### Data analysis

Tanks (n = 3) were used as experimental unit for data on body mineral composition and mineral balance. Individual fish was the experimental unit for data on gene expression (n = 9), on enzyme analyses (n = 9, except for SOD activities, n = 6) and on plasma parameters (n = 6). The analysis of gene expression data was performed after eliminating outliers according to the Grubbs test followed by replacing missing values with estimates using the Yates procedure. Two-way ANOVA was performed after transformation of raw data in ranks to analyse the main effects of the basal diet, micro-mineral premix inclusion and their interactions. The statistical significance was set at P<0.05. In the case of a significant interaction, group means were compared by Tukey’s multiple comparison test as indicated by different superscript letters. All the data analyses were performed using SPSS version 20, IBM Statistics Inc., USA.

## Results

Feed intake and growth-related parameters (Table 4) were significantly higher in groups fed the M vs V diets. No effect of premix supplementation was observed on growth or feed intake. The hepato-somatic index (HSI) was significantly higher in M diet groups. Premix inclusion had an impact on HSI only in the M groups, with M1 fed fish having a higher HSI than M0 fed fish (Table 4). Apparent availability coefficients (AAC, Table 5) of Fe, Cu and Zn were significantly affected by change in basal diet and premix supplementation. AAC of Mn was affected only by change in basal diet, being higher in groups fed diet M. AAC of Se was not different between diets. A significant interaction of premix inclusion with basal diet was observed for AAC of Fe, Zn and Cu, in both cases, premix inclusion decreased the AAC but to a larger extent in the M diet compared with V diet groups. The effect of basal diet on the circulating levels of plasma micro-minerals (Table 6) was significant only for Fe, being higher in V-fed compared to M-fed fish. Premix inclusion did not increase the plasma levels of any of the analysed micro-minerals, except for Mn in M1 vs M0 fed fish. The final whole body concentration of micro-minerals (Table 7) such as Cu, Zn and Se were higher in diet M than in diet V fed fish. Premix inclusion increased the final body concentration of all the analysed micro-minerals, except for Se. Significant interactions between both dietary factors were observed for Mn and Se: premix inclusion increased body Mn concentration in M-fed fish but not V-fed fish. The reverse was observed for Se, where premix inclusion to diet V increased the body Se level, but not in trout fed diet M.

**Table 4:** Growth performance of rainbow trout fed the experimental diets for 12 weeks.

	M0	M1	V0	V1	Basal diet	Premix	Diet x Premix
**FI (g/fish)**	127.6 ± 1.7	131.2 ± 2	113.7 ± 6	115.9 ± 4	< 0.001	0.12	0.76
**FBW (g)**	187.9 ± 6	190.8 ± 6	143.7 ± 13	145.3 ± 6	< 0.001	0.5	0.89
**WG (g)**	168.3 ± 5.3	171.1 ± 4	123.8 ± 12	126 ± 5.4	< 0.001	0.46	0.76
**DGI**	3.6 ± 0.1	3.6 ± 0.04	3 ± 0.15	3 ± 0.07	< 0.001	0.57	0.98
**FE**	1.4 ± 0.03	1.4 ± 0.03	1.2 ±0.04	1.1 ± 0.03	< 0.001	0.54	0.73
**HSI**	1.21 ± 0.2^b^	1.43 ± 0.28^c^	1.05 ± 0.11^a^	1.07 ± 0.13^a^	< 0.001	0.01	0.03

Initial body weight (IBW): 19.8 ± 0.8 g; FBW, final body weight; FI, feed intake; WG, weight gain; Daily growth index, DGI = 100*(FBW^1/3—IBW^1/3)/duration (84 d); FE = Wet weight gain (g)/dry feed intake (g); Hepato-somatic index, HSI = (wet liver weight, g/weight of fish, g)*100. Data are expressed as mean ± SD of n = 3 observations. P-value indicates statistical significance as obtained through two-way ANOVA. In case of a significant interaction, values in the same row with different superscripts are statistically different (P<0.05) as obtained through Tukey’s multiple comparison test.

**Table 5:** Apparent availability coefficient (AAC, %) ofmicro-mineral concentration of rainbow trout.

	M0	M1	V0	V1	Basal diet	Premix	Diet x Premix
**Fe**	39.2 ± 3.1^d^	3.7 ± 0.9^a^	13.4 ± 1.9^c^	10.1 ± 1.2^b^	0.027	0.007	0.012
**Cu**	74.8 ± 0.2^d^	35.1 ± 3.7^c^	39.8 ± 5.5^b^	28.3 ± 4.4^a^	0.014	0.012	0.040
**Mn**	31 ± 4.8^b^	25.7 ± 6.8^b^	7.3 ± 0.4^a^	10 ± 2.6^a^	0.01	0.975	0.349
**Zn**	56.1 ± 7.4^b^	45.8 ± 3^b^	40.2 ± 3^a^	39.4 ± 2^a^	0.011	0.043	0.024
**Se**	80.5 ± 0.7^b^	74.4 ± 1.5^a^	81.4 ± 1.4^b^	79.8 ± 1.7^b^	0.272	0.199	0.238

AAC,% = 100-(100 x((% mineral in faeces)/(% mineral in diet) x (% marker in diet)/(% marker in faeces))).

Data are expressed as mean ± SD of n = 3 observations. P-value indicates statistical significance as obtained through two-way ANOVA. In case of a significant interaction, values in the same row with different superscripts are statistically different (P<0.05) as obtained through Tukey’s multiple comparison test.

**Table 6:** Micro-mineral concentration in the plasma of rainbow trout (μmol L^-1^).

	M0	M1	V0	V1	Basal diet	Premix	Diet x Premix
**Fe**	11.1 ± 2	8.5 ± 2.8	13.6 ± 4.1	14.3 ± 2.9	0.006	0.52	0.23
**Cu**	7.5 ± 2.5	9.5 ± 1.3	10 ± 1.3	8.8 ± 2.2	0.25	0.56	0.72
**Mn**	0.4 ± 0.4^b^	1 ± 0.2^a^	0.9 ± 0.3^a^	0.6 ± 0.1^a^	0.85	0.28	0.01
**Zn**	143.6 ± 27.9	162.3 ± 34	140.4 ± 45.5	150.9 ± 58.4	0.20	0.42	0.40

Data are expressed as mean ± SD of n = 6 observations. P-value indicates statistical significance as obtained through two-way ANOVA. In case of a significant interaction, values in the same row with different superscripts are statistically different (P<0.05) as obtained through Tukey’s multiple comparison test

**Table 7:** Initial and final whole body micro-mineral concentration of rainbow trout (mg kg^-1^ fresh weight).

	Initial	M0	M1	V0	V1	Basal diet	Premix	Diet x Premix
**Fe**	26	14.44 ± 0.23	22.5 ± 5.58	17.9 ± 0.6	21.4 ± 2.5	0.06	0.02	0.38
**Cu**	0.9	0.91 ± 0.12	1.4 ± 0.35	1.9 ± 0.1	2.8 ± 0.2	< 0.01	0.001	0.12
**Mn**	0.9	0.73 ± 0.04^a^	1.2 ± 0.09^b^	1 ± 0.1^b^	0.9 ± 0.1^b^	0.61	0.004	0.002
**Zn**	24.9	15.4 ± 0.51	17.28 ± 0.4	12.1 ± 0.5	13.8 ± 0.6	< 0.001	0.01	0.23
**Se**	0.25	0.3 ± 0.01^b^	0.3 ± 0.005^b^	0.13 ± 0.007^a^	0.16 ± 0.002^a^	< 0.001	0.10	0.03

Data are expressed as mean ± SD of n = 3 observations. P-value indicates statistical significance as obtained through two-way ANOVA. In case of a significant interaction, vvalues in the same row with different superscripts are statistically different (P<0.05) as obtained through Tukey’s multiple comparison test.

Data on the activity of the analysed enzymes is presented in Table 8. Of the two apical metal reductases assayed in intestine and liver, only ferric reductase activity in the intestine was significantly affected by the change in basal diet, being 2-fold higher in fish fed the V diet. Among the different metalo-enzymes analysed, only the activity of hepatic Se-dependent GPX was differentially regulated, being higher in liver of fish fed M diet. Premix inclusion did not affect the activity of any of the analysed enzymes.

**Table 8:** Analysed activities of selected enzymes involved micro-mineral absorption, transport or metabolism (unit per mg protein).

	Tissue	M0	M1	V0	V1	Basal diet	Premix	Diet x Premix
Ferric reductase	Intestine	0.71 ± 0.33	0.8 ± 0.36	1.87 ± 0.66	1.89 ± 0.89	0.001	0.81	0.88
	Liver	3.52 ± 0.94	4.17 ± 3.25	6.40 ± 4.73	3.59 ± 1.31	0.32	0.35	0.14
Cupric reductase	Intestine	189 ± 43	200 ± 66	180 ± 39	190 ± 46	0.61	0.59	0.98
	Liver	94 ± 23	91 ± 5.9	76.8 ± 10.6	94.1 ± 12.1	0.20	0.19	0.07
ALP	Intestine	455 ± 149	444 ± 246	383 ± 119	410 ± 90	0.38	0.89	0.76
	Liver	222 ± 41	221 ± 101	182 ± 53	208 ± 66	0.32	0.63	0.61
Total SOD	Liver	102 ± 15	99 ± 8	109 ± 13	97 ± 12	0.68	0.35	0.38
CuZn-SOD	Liver	40.0 ± 12.9	41.2 ± 22.3	43.7 ± 14.5	40.1 ± 15.2	0.88	0.89	0.78
Mn-SOD	Liver	57.3 ± 15.8	61.6 ± 4.2	56.9 ± 5.4	64.9 ± 10.1	0.41	0.21	0.99
Catalase	Liver	969 ± 156	975 ± 297	1023 ± 190	1064 ± 219	0.39	0.78	0.83
Se-GPX	Liver	37.0 ± 17.5	49.4 ± 15.7	31.7 ± 3.6	31.4 ± 7.3	0.02	0.20	0.17

ALP, Alkaline phosphatase; SOD, Superoxide-dismutase; GPX, Glutathione peroxidase; Data presented as mean ± SD of n = 9 samples for ferric reductase, cupric reductase, alkaline phosphatase, catalase and glutathione peroxidase; for the three SOD enzymes namely totalSOD, Cu-Zn-SOD and Mn-SOD, n = 6. P-value indicates statistical significance as obtained through two-way ANOVA.

**Fig 1:**
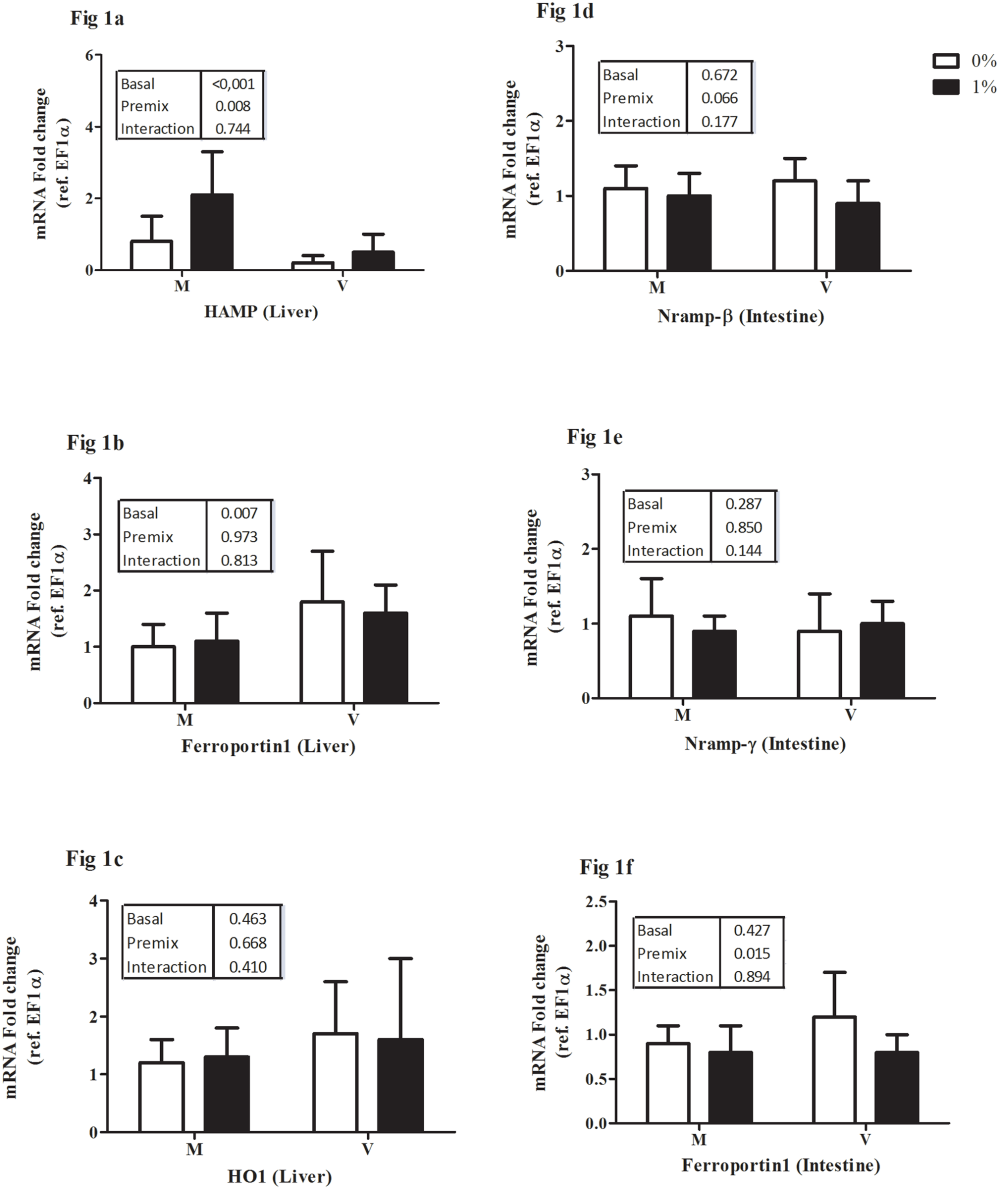
Transcription of selected transporters and regulators of iron metabolism in the liver and intestine of rainbow trout expressed relative to elongation factor 1alpha (EF1a). (i) Liver: Hepcidin (HAMP, Fig 1a); Ferroportin 1 (FPN1, Fig 1b); Heme-oxygenase 1 (HO1, Fig 1c). (ii) Intestine: Nramp-β, (Fig 1d) and Nramp-γ (Fig 1e); Ferroportin 1 (Fig 1f). M, marine ingredient based diet and V, vegetable ingredient based diet; white bars, un-supplemented diet (0%); black bars, and premix supplemented diet (1%). Each bar represents mean ± SD of n = 9 samples. P-values obtained from two-way ANOVA on the main effects of basal diet, premix inclusion and interaction are provided in insets. Different superscripts indicate significant difference in case of a significant interaction (Tukey’s multiple comparison test).

**Fig 2:**
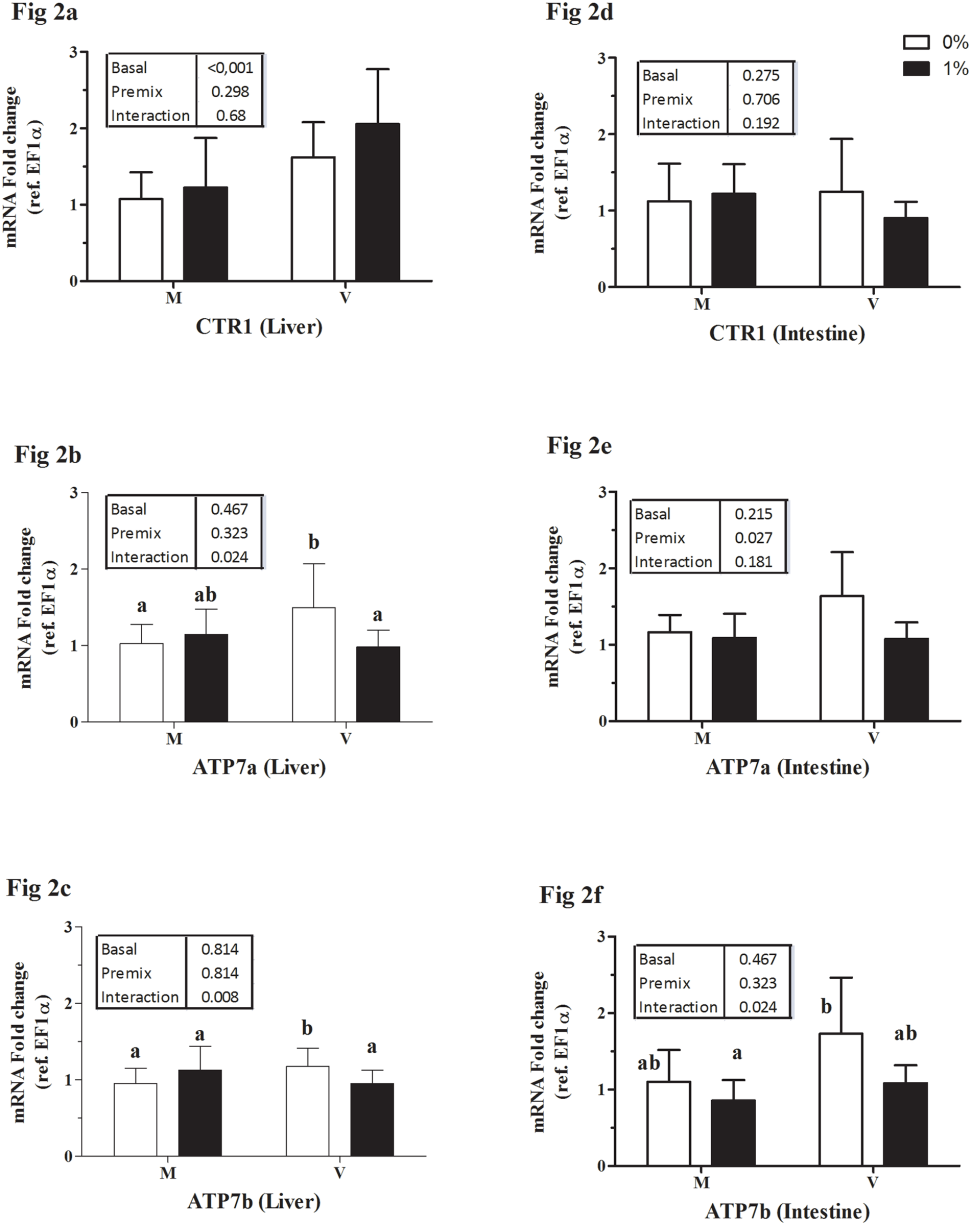
Expression of intestinal and hepatic copper transporters expressed relative to elongation factor 1alpha (EF1a). Liver: copper transporter1 (CTR1, Fig 2a); ATP7a (Fig 2b) and ATP7b (Fig 2c). Intestine: copper transporter1 (CTR1, Fig 2d); ATP7a (Fig 2e) and ATP7b (Fig 2f).M, marine ingredient based diet and V, vegetable ingredient based diet; white bars, un-supplemented diet (0%); black bars, premix supplemented diet (1%). Each bar represents mean ± SD of n = 9 samples. P-values obtained from two-way ANOVA on the main effects of basal diet, premix inclusion and interaction, if any are provided in insets. Different superscripts indicate significant difference in case of a significant interaction (Tukey’s multiple comparison test).

**Fig 3:**
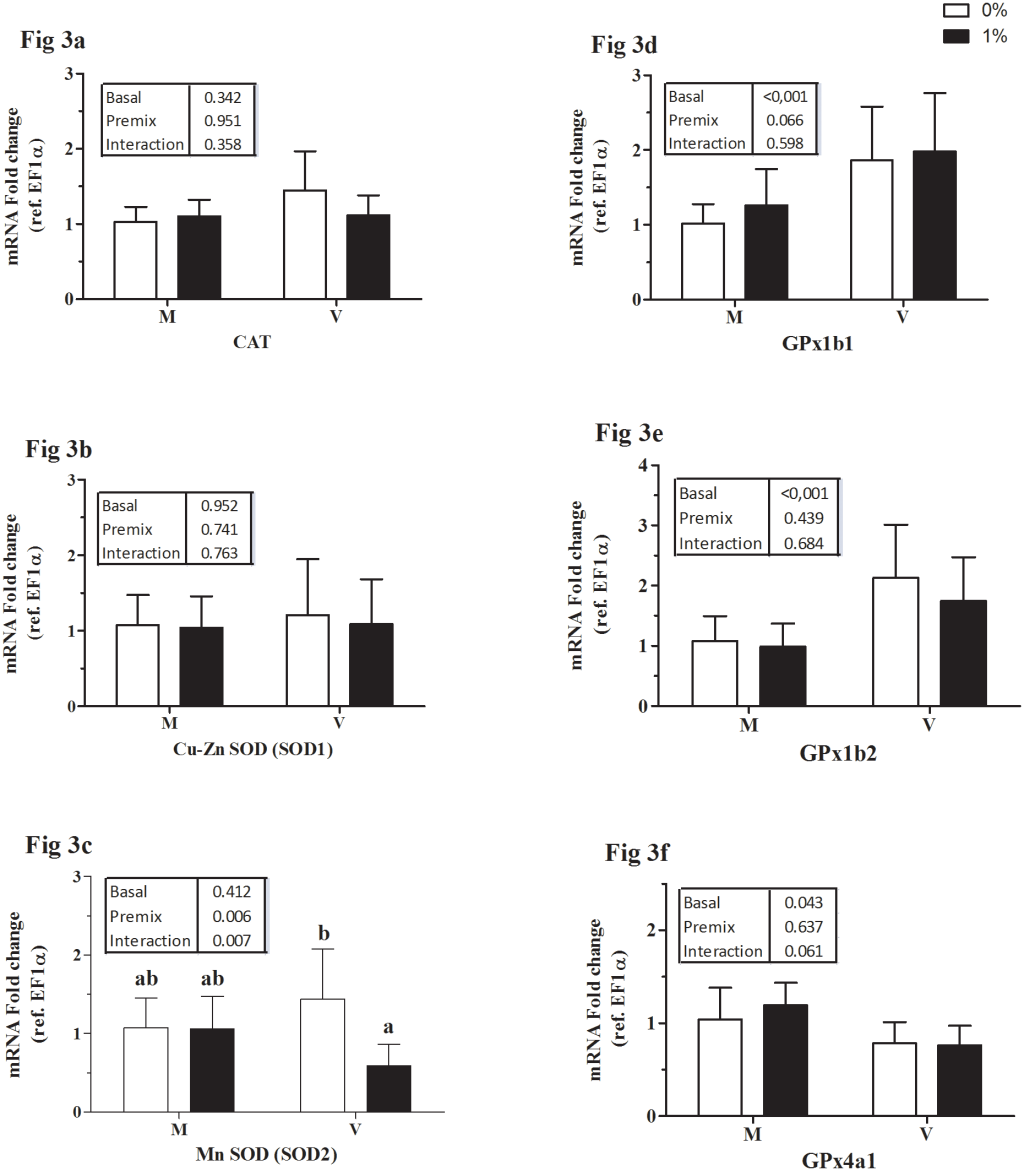
Transcription of metalo-enzyme genes in liver expressed relative to elongation factor 1alpha (EF1a). Catalase (CAT, Fig 3a); Cu-Zn superoxide dismutase, (Cu-Zn SOD, Fig 3b); Mn superoxide dismutase, (Mn-SOD, Fig 3c); Glutathione peroxidase, GPx1b1 (Fig 3d); GPX1b2 (Fig 3e) and GPX4a1 (Fig 3f). M, marine ingredient based diet and V, vegetable ingredient based diet; white bars, un-supplemented diet (0%); black bars, premix supplemented diet (1%). Each bar represents mean ± SD of n = 9 samples. P-values obtained from two-way ANOVA on the main effects of basal diet, premix inclusion and interaction, if any are provided in insets. Different superscripts indicate significant difference in case of a significant interaction (Tukey’s multiple comparison test).

Data on relative mRNA expression of genes involved in the transport and metabolism of the studied micro-minerals are presented in Figs 1 to 3: Fe (Fig 1), Cu (Fig 2), and Mn, Zn, Se together (Fig 3).

(i) Iron: In the liver of trout, HAMP expression was increased by feeding diet M and by premix inclusion, in particular in diet M1 group (Fig 1a). The expression of FPN1 (Fig 1b) was higher in fish fed the V vs M diet without effect of premix inclusionwhile HO1 expression was unaffected (Fig 1c). In the intestine, the expression of DMT1 isoforms namely, Nramp-β (Fig 1d) and Nramp-γ (Fig 1e), were not significantly different among the groups. Feeding diets with the inclusion of premix reduced the expression of intestinal FPN1 (Fig 1f). (ii) Copper: In liver, the basal V diet increased CTR1 expression (Fig 2a). Hepatic expression of ATP7a was higher in V0 fed fish than in those fed diets V1 or M0 (Fig 2b); whereas that of ATP7b was slightly higher in the V0 groups (Fig 2c). Intestinal expression of CTR1 was not different between the groups (Fig 2d). The intestinal expression of Cu-transporting P-type ATPase, ATP7a was reduced by premix inclusion (Fig 2e). Intestinal expression of ATP7b was higher in V0-fed fish compared to M1-fed fish (Fig 2f). ATOX1 expression in both intestine and liver was not significantly different between the groups (data not presented). (iii) Metalo-enzymes: Hepatic gene expression of catalase (CAT; Fig 3a) and Cu-Zn superoxide-dismutase (SOD1; Fig 3b) were not statistically different between the treatments. Hepatic expression of Mn-SOD (SOD2) strongly decreased by premix inclusion to basal V diet, being the lowest in liver of the V1 fed group (Fig 3c). Expression of GPX1b1 (Fig 3d) and GPX1b2 (Fig 3e) was higher in liver of fish fed the V than the M diet, whereas the expression of GPX4a1 (Fig 3f) was lower in liver of V vs M fed fish.

## Discussion

The growth performance of the trout fed the all plant based diets in this study was considerably lower than when fed the fish meal based diets, as observed in our earlier studies [19,20]. In contrast with the latter study where fish were fed fixed rations of the plant- and fishmeal-based diets [20], decreased energy intake in the V vs M dietary group may partially explain the lower growth in the present study as feed intakes were lower in V-groups. The lack of a positive effect of adding the micro- mineral premix on growth performance indicates that the micro-mineral supply from the un-supplemented basal diets was sufficient to support good growth of the trout in both diet groups. Effects of the change in basal diet, the premix inclusion and the interaction between the two variables on apparent availability, plasma and whole body mineral levels, activity of brush border apical reductases and expression of genes involved in Fe and Cu metabolism and on hepatic activity and expression of metalo-enzymes containing Fe, Cu, Mn, Zn or Se are discussed in the sections to follow.

### Iron: Intestinal absorption and hepatic metabolism

Change in basal diet and premix inclusion had significant effects on the analysed markers of Fe absorption and metabolism in rainbow trout. The proportion of Fe absorbed from the diet, which is the major source of Fe to fish [27,28], has been found to decrease with increasing dietary levels [8], as reflected here by the lowered Fe AAC values following premix addition. Divalent metal transporters (DMT1 or Nramp) are known to facilitate cellular transport of Fe^2+^across intestinal apical membranes [29] and a brush border ferric reductase (FR) activity exists for the reduction of non-heme Fe^3+^ to Fe^2+^, the substrate for DMT1 [30,31]. The activity of intestinal FR also indicated that the available Fe supply was higher from M than from V diets and that, as in mammals, FR independent mechanism might exist for heme-Fe uptake in rainbow trout when fed fish meal based diets. In trout, gastro-intestinal expression of Nramp (DMT) genes were initially induced at day7 on exposure to high dietary Fe but decreased at day14 [32]. Such a phenomenon may explain the lack of differential expression in intestinal DMT isoforms (Nramp-β and Nramp-γ) after 12 weeks of feeding in the present study. Moreover, DMTs are also transporters of several other divalent metal ions, and interactions with Cu^++^ or Zn^++^were shown to exist in the apical uptake of Fe by DMTs in trout intestine [30,33].

Given this non-specific nature of DMTs, it is likely that a more specific and critical step exists in regulating intestinal Fe absorption. In this respect, Kwong et al. [32] emphasized the need for better understanding the role and physiological importance of ferroportin (FPN1) and hepcidin (HAMP) in systemic Fe regulation in fish. In vertebrates, ferroportin (FPN1) expression is considered vital for the cellular export of Fe [34] and hepcidin (HAMP) exerts an inhibitory action on Fe export by FPN1 [35]. In mammals and also in zebrafish, ferroportin assisted basolateral extrusion of Fe was identified as the rate limiting step of intestinal iron absorption [13,36]. Hepcidin is encoded by HAMP gene in liver of mammals as also documented in some teleosts [37], including rainbow trout [38]. The trout in our study fed the V diet had lower expression of hepcidin (HAMP) together with increased expression of FPN1 in the liver, as expected from the inverse relationship between HAMP and hepatic FPN1 in mammals [38]. Premix inclusion on the other hand up-regulated HAMP expression in liver, but did not alter the expression of hepatic FPN1.

Like in mammals, body iron stores in fish are known to regulate intestinal Fe absorption and homeostasis [8]. Experimental Fe overload has been reported to increase liver HAMP expression in mammals [39] and in fish [14]. In the same line, Yeh et al. [40] reported Fe overload in rats through suppression of hepcidin (HAMP), which in turn induced over-expression of intestinal FPN1. Also here, transcript levels of hepatic HAMP and of FPN1 in trout intestine also showed an opposite response to the premix inclusion, whereas intestinal FPN1 expression remained unaffected by the basal diet composition. Cooper et al. [31] reported a biphasic pattern of Fe accumulation in the intestinal epithelia of gulf toad fish, suggestive of the periodic partial loss of dietary Fe due to epithelial sloughing. Rainbow trout also showed accumulation of dietary Fe in the intestine [32]. The enterocytes of the intestinal epithelium are sloughed off and replenished regularly, at 2–3 day intervals in mammals and probably few weeks in the case of fish [41]. Consequently, the Fe accumulated in the intestinal epithelium will not be absorbed and end up in the faeces. The high liver HAMP expression in the M1 group possibly explains the low AAC of Fe in these fish, which is however not reflected here in changes in expression of FPN1, affected by premix supplementation but not by basal diet. Our results suggest that systemic Fe homeostasis in rainbow trout could be under transcriptional regulation of hepcidin and ferroportin, but as suggested by Kwong et al. [32], further studies are required to better understand the mechanism.

### Copper: Intestinal and hepatic transport

Copper uptake, transport and homeostasis in mammals are mediated by a high affinity copper transporter (CTR1), Cu chaperons (ATOX1 and CCS) and P-type Cu-transporting-ATPases (ATP7a and ATP7b) [42]. The existence of a high-affinity Cu transporter in the gastrointestinal tract with conserved regions for cellular Cu transport has been characterised in zebra fish [43], gilthead seabream [15] and rainbow trout [44]. In the intestine, ATP7a is believed to be involved in the basolateral transfer of Cu [45]. Gastrointestinal expressions of CTR1 in rainbow trout [44], and of CTR1 and ATP7a in gilthead sea bream [15,16] were down-regulated by high dietary Cu supply. In the present study, AAC of Cu and intestinal expression of ATP7a was down-regulated by inclusion of premix. On the other hand, fish fed the V diets showed elevated hepatic CTR1 expression and had high body Cu, presumably due to Cu accumulation in the liver. In stickleback, exposure to Cu in the aquatic environment has been found to alter the transcription of the rate limiting enzymes in cholesterol and bile acid synthesis [46]. In Atlantic salmon, plant-ingredients, more specifically soybean meal, also disturb hepato-biliary functioning and cholesterol biosynthesis [47]. As such, the high whole body Cu in the fish fed the plant-based V diet (in the present and in previous study [19]), could be a result of impaired hepato-biliary Cu excretion leading to Cu accumulation in the body.

### Activity and expression of metalo-enzymes

Activity and gene expression of metalo-enzymes have been used as biomarkers of micro- mineral status in mammals and fish. Catalase (CAT) activity and expression decreases with a dietary deficiency in Fe supply in rabbits [48]. Alkaline phosphatase has been used as a marker for Zn status in several fish species including Atlantic salmon [49] and rainbow trout [50], where the activity is reduced during Zn deficiency. No such effect was observed in the present study suggesting that dietary Fe and Zn supply was not deficient even in the un-supplemented diets. Low dietary supply of Mn results in low whole body Mn-status in rainbow trout [51] and Atlantic salmon [52], as observed for whole body-Mn and plasma Mn levels in the present study, with M0 fed fish. In fish, hepatic MnSOD (SOD2) activity has been found to be reduced at high dietary supply of Mn [53,54]. In the present study, the mineral premix down regulated MnSOD transcription but only in the V1 versus V0 group, likely due to the inherently high level of Mn already present in diet V0, while the activity was unaffected. Together, our results might indicate insufficient supply of Mn in M0 diet and an excess Mn supply with V1 diet. With regard to Se, the lower activity of Se-dependent glutathione peroxidase in V vs M-diet groups might be due to the low Se content of V diets. At the transcriptional level, we indeed found reduced expression of GPX4a1 in the V-diet groups, whereas that of other GPX1 isoforms increased. According to Pacitti et al. [55], among the different GPX transcripts described in rainbow trout, GPX1 isoforms are the most sensitive, though changes in transcription did not coincide with those in enzyme activity. Similarly, in the present study, the response in expression of GPX1 was not in line with the measured GPX activity. Moreover, as no effect of premix supplementation was observed, it is possible that the changes in the expression GPX isoforms are influenced by other unknown factors. On the whole, based on the results and the recommended levels for rainbow trout by NRC [17,18], except for Mn in M diet and Se in V diets, the basal dietary supply of other micro- minerals was sufficient to meet the normal physiological needs of rainbow trout.

## Conclusion

The present results suggest that regulation of micro-mineral absorption and metabolism in rainbow trout depends on the dietary ingredient composition (fish- versus plant-ingredient based diets) and on the inclusion of mineral premix. We also found that the dietary ingredient composition interacts with premix supplementation, especially for Fe and Cu. Further detailed investigations are required to better understand such interactions and the cellular and molecular regulation of micro-mineral status in rainbow trout as affected by dietary factors.
